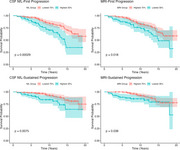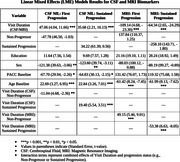# Evaluating CSF NfL and Hippocampal Atrophy as Predictors of Dementia Progression: A 20‐Year Longitudinal Study

**DOI:** 10.1002/alz70856_105145

**Published:** 2026-01-08

**Authors:** Ramkrishna Kumar Singh, Semere Bekena, Yiqi Zhu, Nikitha Damera, Kaylin Taylor, Jean‐Francois Trani, Ganesh M. Babulal

**Affiliations:** ^1^ Washington University School of Medicine, Saint Louis, MO, USA; ^2^ Washington University School of Medicine, St. Louis, MO, USA; ^3^ Washington University, St. Louis, MO, USA; ^4^ Natiobal conservatory of Arts and Crafts, Paris, NA, France; ^5^ University of Johannesburg, Johannesburg, Gauteng Province, South Africa; ^6^ Knight Alzheimer Disease Research Center, St. Louis, MO, USA

## Abstract

**Background:**

Neuroinflammation is a key risk factor in dementia pathogenesis. Biomarkers such as cerebrospinal fluid (CSF), neurofilament light (NfL), and MRI‐derived hippocampal volume provide insights into early neurodegeneration. This study assessed their utility in predicting cognitive decline and as tools for early dementia screening.

**Method:**

This 20‐year longitudinal follow‐up (2003–2023) included 279 cognitively normal participants aged 55 and older, with annual visits, using data from the Knight ADRC cohort in St. Louis, USA. Baseline CSF NfL, MRI hippocampal volume, and Clinical Dementia Rating (CDR) were assessed. The first progression was defined as a single transition from CDR 0 to 0.5, and sustained progression required consecutive CDR scores ≥ 0.5. Kaplan‐Meier curves, Cox models, and linear mixed‐effects (LME) models evaluated biomarker associations with dementia progression and longitudinal changes.

**Result:**

At baseline, the mean age of the participants was 66.5 years (SD: 6.08), and 58.4% were female. The highest 30% of CSF NfL levels had a mean of 973 pg/mL. The lowest 30% of hippocampal volumes had a mean of 6310 mm^3^. Over a mean follow‐up of 11.41 years (SD: 3.5), 73 participants (26%) experienced first progression and 35 (13%) sustained progression. Individuals with high CSF NfL levels showed significantly faster time to both first progression (*p* = 0.00029) and sustained progression (*p* = 0.0075), as shown by the Kaplan‐Meier curves (Figure 1). Cox models showed that high CSF NfL levels were associated with an increased risk of first progression (HR: 1.83, 95% CI: 1.11–3.01; *p* =  0.018) but not with sustained progression (HR: 1.83, 95% CI: 0.90–3.69; *p* =  0.093). No significant associations were observed between hippocampal volume and the risk of either progression. LME models showed faster increases in CSF NfL (*p* < 0.001) and steeper declines in hippocampal volume (*p* < 0.001) in both progressor groups compared to non‐progressors (Table 1).

**Conclusion:**

Longitudinal CSF NfL and hippocampal volume are reliable biomarkers for predicting cognitive decline. They have the potential to identify at‐risk adults aged 55 and older early and effectively monitor the progression of dementia.